# Optical and Structural Analysis of TiO_2_–SiO_2_ Nanocomposite Thin Films Fabricated via Pulsed Laser Deposition Technique

**DOI:** 10.3390/nano13101632

**Published:** 2023-05-13

**Authors:** Laid Kadri, Abdelkader Abderrahmane, Georgiana Bulai, Aurelian Carlescu, Corneliu Doroftei, Iuliana Motrescu, Silviu Gurlui, Liviu Leontie, Mohamed Adnane

**Affiliations:** 1 Department of Sciences and Technology, Faculty of Sciences and Technology, University Ahmed Draia of Adrar, Adrar 01000, Algeria; laid.kadri@univ-adrar.edu.dz; 2 Department of Electrical Engineering, Chosun University, 375, Seosuk-dong, Dong-gu, Gwangju 501-759, Republic of Korea; abderrahmane.abdelkader@gmail.com; 3 Laboratoire de Structure, Elaboration et Application des Matériaux Moléculaires (SEA2M), Université de Mostaganem Abdelhamid Ibn Badis, B.P. 227, Mostaganem 27000, Algeria; 4 Integrated Center for Studies in Environmental Science for The North-East Region (CERNESIM), Department of Exact Sciences, Institute of Interdisciplinary Research, Alexandru Ioan Cuza University of Iași, 700506 Iași, Romania; georgiana.bulai@uaic.ro (G.B.); aurelian.carlescu@uaic.ro (A.C.); corneliu.doroftei@uaic.ro (C.D.); 5 Department of Exact Sciences & Research Institute for Agriculture and Environment, Iasi University of Life Sciences, 3 Sadoveanu Alley, 700490 Iasi, Romania; imotrescu@uaiasi.ro; 6 Faculty of Physics, LOA-SL, Alexandru Ioan Cuza University of Iasi, Bulevardul Carol I, Nr. 11, 700506 Iasi, Romania; 7 Faculty of Physics, Alexandru Ioan Cuza University of Iasi, Bulevardul Carol I, Nr. 11, 700506 Iasi, Romania; 8 Laboratoire de Microscopie Electronique et Sciences des Matériaux (LMESM), Département de Technologie des Matériaux, Faculté de Physique, Université des Sciences et de la Technologie d’Oran Mohamed Boudiaf (USTO-MB), El M’naouar BP 1505 Bir El Djir, Oran 31000, Algeria; mohamed67adnane@gmail.com

**Keywords:** TiO_2_–SiO_2_, nanocomposite thin films, pulsed laser deposition, structural properties, optical properties, UV–vis transmittance spectra

## Abstract

TiO_2_–SiO_2_ nanocomposite thin films have gained the attention of the scientific community due to their unique physical and chemical properties. In this paper, we report on the fabrication and characterization of a TiO_2_–SiO_2_ nanocomposite disk-shaped target. The target was used for the deposition of TiO_2_–SiO_2_ nanocomposite thin films on fluorine-doped tin oxide/glass substrates using the pulsed laser deposition (PLD) technique. The thicknesses of the thin films were fixed to 100 nm, and the deposition temperature ranged from room temperature to 300 °C. As revealed by the microstructural and morphological characterizations revealed, the TiO_2_–SiO_2_ nanocomposite thin films are amorphous and display homogeneous distribution. The determined values of the indirect optical band gap range from 2.92 to 3.07 eV, while those of the direct optical band gap lie between 3.50 and 3.55 eV. Additionally, as the deposition temperature decreases, the light transmission increases in the visible and in the ultraviolet ranges, which is suitable for flexible perovskite solar cells. This research can uncover new insights into the fabrication of amorphous TiO_2_–SiO_2_-based nanostructured thin films using the PLD technique for perovskite solar cell technology.

## 1. Introduction

Titanium dioxide (TiO_2_) and silicon dioxide (SiO_2_) are semiconductor materials that are widely studied as thin films due to their unique properties. On one hand, TiO_2_ has a wide bandgap of 3.2 eV in its anatase phase and 3.0 eV in its rutile phase [1,2], a high refractive index [3], and excellent photocatalytic activity, making it a good choice for optical applications, especially solar cells [4]. Amorphous TiO_2_ thin films can have smooth surface morphologies and optical properties similar to crystalline anatase TiO_2_ owing to their similar electronic structures [2]. On the other hand, SiO_2_ exhibits exceptional mechanical properties and excellent chemical and thermal stabilities, making it ideal for applications that require high durability and reliability [5,6]. Moreover, SiO_2_ is used as a dielectric material in microelectronic circuits. Several techniques have been used to deposit TiO_2_ and SiO_2_ thin films, including chemical vapor deposition (CVD) [7,8], molecular beam epitaxy [9,10], plasma-enhanced chemical vapor deposition [11,12], the sol–gel process [13,14], and pulsed laser deposition (PLD) [15,16], etc. PLD is a physical vapor deposition technique that uses high-energy laser pulses to ablate the target’s material to obtain, via vapor condensation on a substrate, thin films with excellent structural and optical properties. This technique is powerful and versatile, allowing for the precise control of the thin film’s thickness, stoichiometry, and composition, making it an excellent method for the synthesis of complex structures and composites [17,18,19]. 

The TiO_2_–SiO_2_ nanocomposite thin films display better characteristics when compared to individual TiO_2_ and SiO_2_ materials. In fact, the combination of TiO_2_ and SiO_2_ results in enhanced mechanical, thermal, and optical properties [20]. TiO_2_–SiO_2_ nanocomposite films exhibit enhanced performance in terms of charge transport and stability, making them particularly suitable for electron transport layers (ETLs) in perovskite solar cells (PSCs) [21,22]. The design and development of TiO_2_–SiO_2_ nanocomposite thin films is a very promising research topic that could lead to development of more efficient and stable PSCs. In addition, TiO_2_–SiO_2_ amorphous films cover a wide range of possible refractive indices, and they exhibit a high dielectric constant and a low leakage current [23]. For instance, a study by Zhao et al. [24] reported the synthesis of TiO_2_–SiO_2_ hybrid films with a tunable refractive index using the sol–gel technique. Another study by Mitronika et al. [25] investigated the effect of SiO_2_ content on the optical and electrical properties of TiO_2_–SiO_2_ nanocomposite thin films in which they showed that the best compromise results in terms of dielectric permittivity improvement, charge injection, and transport behavior were obtained in the case of a Ti_0.33_Si_0.66_O_2_ nanocomposite. Wang et al. [26] reported that a value of x = 0.5 in the Ti_x_Si_1-x_O_2_ nanocomposite results in the highest average transmittance. However, there are very few studies that have demonstrated the successful fabrication of TiO_2_–SiO_2_ composite thin films using pulsed laser deposition [27], and there are still some limitations that must be addressed, such as the optimization of the parameters and deposition conditions, as well as the identification of the best composition and structure for TiO_2_–SiO_2_ nanocomposite thin films.

In this paper, we report the fabrication of a TiO_2_–SiO_2_ nanocomposite target and investigate its morphological and microstructural properties. Raman spectroscopy and X-ray diffraction (XRD) analyses confirmed the presence of two phases, anatase (TiO_2_) and quartz (SiO_2_), in the target. We used the PLD technique to develop TiO_2_–SiO_2_ nanocomposite thin films. The microstructural characterization of the nanocomposite thin films deposited on a fluorine-doped tin oxide (FTO)/glass substrate showed that the FTO grains were overlaid by 100 nm thick nanocomposite thin films. An analysis of their optical properties revealed that the nanocomposite nanofilms exhibit indirect optical band gaps of 3.00 eV, 3.06, 3.07, and 2.92 eV for thin films deposited at 25 °C, 200 °C, 250 °C, and 300 °C, respectively. The direct optical band gaps were found to be equal to 3.50 eV, 3.53, 3.55, and 3.51 eV for thin films deposited at 25 °C, 200 °C, 250 °C, and 300 °C, respectively. The purpose of this research is to investigate the properties of TiO_2_–SiO_2_ nanocomposite thin films for perovskite solar cell applications. The findings of this study will contribute to the development of TiO_2_–SiO_2_ nanocomposite thin films for the next generation of optoelectronics, photonics, and photovoltaics.

## 2. Materials and Methods

### 2.1. Target Preparation

The oxide powders TiO_2_ (BIOCHEM Chemopharma, Cosne-Cours-sur-Loire, France; product code: 320150500) and SiO_2_ (Kojundo Chemical Laboratory Co., Ltd., Tokyo, Japan; SiO_2_ quartz), were mixed at a 2.2 weight ratio of TiO_2_ to SiO_2_ and dried at 100 °C. Then, the mixture was ground in an agate mortar and pressed into a disk shape with a 13 mm diameter and a thickness of 3 mm. The target was sintered at temperature of 800 °C for 4 h with a ramp rate of 10 °C/min.

### 2.2. Substrate Preparation

FTO/glass substrates (10 × 10 × 3.1 mm^3^; sheet resistance: ~7 Ω/sq) were used in this study. The thickness of the FTO layer was 600 nm. The substrates were cleaned successively in ethyl alcohol, acetone, and deionized water for 10 min each. Finally, the substrates were dried with air at room temperature. 

### 2.3. Deposition Parameters

TiO_2_–SiO_2_ nanocomposite thin films were deposited on an FTO/glass substrate using the PLD method. An Nd:YAG laser with a 2nd harmonic and a wavelength of 532 nm was used, and the pulse duration was fixed at 10 ns with a repetition rate of 10 Hz; the pulse energy was 50 mJ/pulse, and the spot size was approximately 2.5 mm². The laser fluence and deposition pressure were set to 2.0 J/cm² and 50 mTorr, respectively. The target-to-substrate distance was fixed at 50 mm, and 10 min was chosen as the deposition time. Finally, four deposition temperatures were chosen: 25 °C, 200 °C, 250 °C, and 300 °C.

### 2.4. Thin Film Characterizations

The thicknesses of the as-deposited TiO_2_–SiO_2_ nanocomposite thin films were determined to be 100 nm in all samples through the use of a Dektak XT stylus profilometer (Bruker, Paris, France). Morphological characterizations of the target and the films were performed using a scanning electron microscope (SEM) (Quanta 450, FEI, Thermo Fisher Scientific, Hillsboro, OR, USA) coupled with an energy dispersive X-ray detector (EDS) (EDAX, AMETEK Inc., Berwyn, PA, USA). The EDS analysis was performed using the TEAM version V4.1 system (EDAX Inc.); calibration was carried out using a standard AlCu sample (Cu foil on an Al grid). In map analysis mode, an average number of 50,000 frames was used with a dwell of 200 ms. The XRD patterns with 2*θ* ranged from 20 to 80 ° and were obtained using a Shimadzu LabX XRD-6000 diffractometer (Shimadzu, Kyoto, Japan) with CuK*α* radiation (*λ* = 1.54 Å). Raman spectroscopy measurements were performed using an InVia confocal Raman Microscope (Renishaw, Wotton-under-Edge, UK). We used the open-source software Image J, version 1.53t, for the particle size distribution determination from the SEM micrographs of the target FTO/glass substrate and the TiO_2_–SiO_2_ nanocomposite thin films. Finally, an Avantes UV–vis spectrometer (AvaSpec 2048, Schiphol, The Netherlands) was used for the optical characterizations. The optical bandgaps (*E_g_*) of the nanocomposite thin films were determined from the absorption spectra via Tauc plots.

## 3. Results and Discussion 

### 3.1. Nanocomposite Target Properties

**Figure 1:**
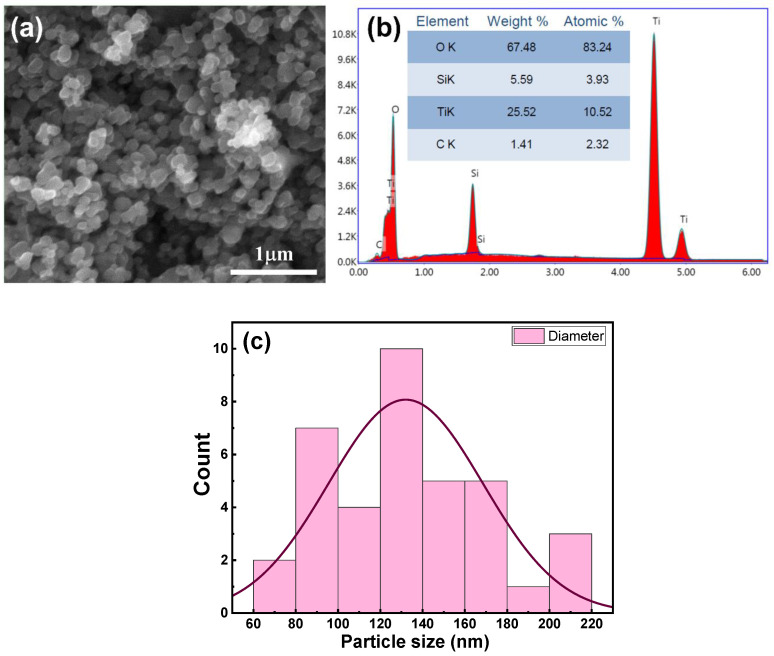
(**a**) SEM image of sintered TiO_2_–SiO_2_ nanocomposite target, (**b**) EDX spectrum of target, and (**c**) the particle size distribution histogram deduced from the SEM image.

Figure 1a presents an SEM image of the fabricated target, obtained at an acceleration voltage of 20 kV; the scale of the image is 1μm. As can be seen from the image, the nanoparticles were homogeneously dispersed, and the TiO_2_ and SiO_2_ phases cannot be distinguished. The EDX analysis of the sintered TiO_2_–SiO_2_ nanocomposite target is represented in Figure 1b. The nanocomposite can be denoted by Ti_x_Si_1−x_O_y_, where x and y correspond to the Ti and O contents, respectively. Values of x and y can be determined from the atomic contents of titanium [Ti] and silicon [Si] using the following equation [25]:

(1) $$x=\frac{\left[Ti\right]}{\left[Ti\right]+\left[Si\right]}$$

According to Equation (1), x was determined to be approximately 0.72; therefore, our nanocomposite had the following composition: Ti_0.72_Si_0.28_O_y_. We note that the EDX analysis was carried out on the sintered TiO_2_–SiO_2_ nanocomposite target after it was used for the deposition of the thin film, and we found that carbon was present as an impurity even after cleaning the target surface, as can be seen in the inset of Figure 1b. The average particle size in the target was determined from the SEM images to be 132 nm, as shown in Figure 1c. The relatively large size of the nanoparticles can be attributed to the limitations of the mechanical grinding method used for target preparation.

**Figure 2:**
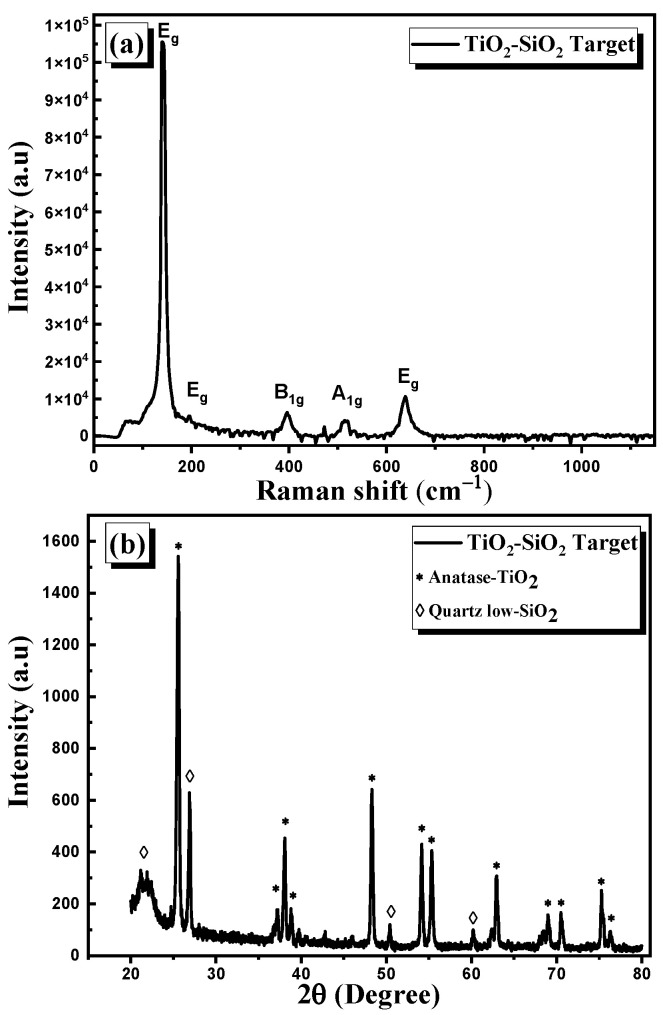
(**a**) Raman spectra and (**b**) XRD pattern of sintered TiO_2_–SiO_2_ nanocomposite target.

The Raman spectra of the sintered TiO_2_–SiO_2_ nanocomposite target are presented in Figure 2a. The target exhibits peaks at 142.2, 195.9, 395.1, 513.6, and 637.5 cm^−1^ which belong to the TiO_2_ in its anatase form [28,29]. The peaks correspond to the *E_g_*, *E_g_*, *B_1g_*, *A_1g_*, and *E_g_* modes, respectively. It should be noted that the peaks corresponding to the rutile TiO_2_ are absent. The XRD diagram of the target is shown in Figure 2b. It reveals that the diffraction peaks correlate well with anatase TiO_2_ according to Anatase—TiO_2_—PDF 98-002-4276, tetragonal, and with quartz SiO_2_ according to Quartz low—SiO_2_—PDF 98-006-2406, hexagonal. High-purity TiO_2_ anatase phase is confirmed, and there was a good agreement between the Raman spectroscopy and XRD results. However, the presence of SiO_2_ quartz in the target was detected only from the analysis of the XRD pattern. The absence of a Raman shift corresponding to SiO_2_ may have been caused by the low Raman signal of SiO_2_ nanoparticles.

### 3.2. Nanocomposite Thin Films Characterizations 

#### 3.2.1. SEM Morphological Analysis

**Figure 3:**
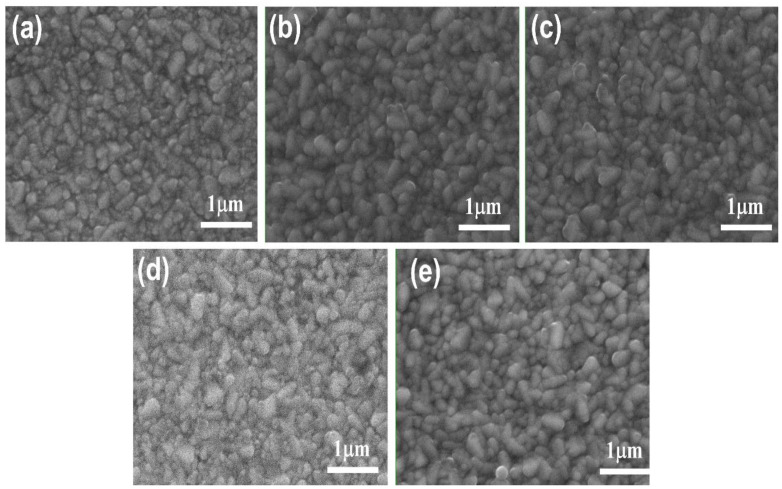
SEM top view images of (**a**) FTO/glass substrate; TiO_2_–SiO_2_ nanocomposite thin films deposited at (**b**) 25 °C, (**c**) 200 °C, (**d**) 250 °C, and (**e**) 300 °C.

The SEM images of the FTO/glass substrate and the nanocomposite thin films deposited on FTO/glass substrates at different temperatures are shown in Figure 3. As can be seen in Figure 3a, the FTO displays irregularly shaped grains. After the deposition of the thin film, the FTO grains remained visually recognizable; however, they transitioned from sharp and angular shapes into smoother and not-sharp shapes, as can be seen in Figure 3b–e, confirming that the FTO grains were overlaid by the nanocomposite thin films. Similar behavior was reported in the work of Z. Jiang et al. [30] in which the surface morphology of an FTO substrate changed from a pyramidal morphology to a non-stereotypical morphology after the deposition of TiO_2_ thin films via magnetron sputtering. Su et al. [31] reported the overlaying of FTO irregular grains with TiO_2_ nanohole array thin films obtained via the electrodeposition technique. A high degree of homogeneity of the composition was confirmed by the absence of a clear distinction between the TiO_2_ and SiO_2_ nanoparticles.

**Figure 4:**
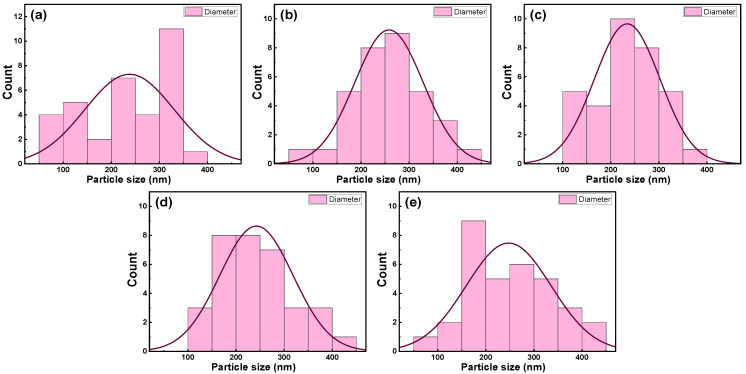
Particles size distribution histogram determined from SEM image of (**a**) an FTO/glass substrate and TiO_2_–SiO_2_ nanocomposite thin films deposited on FTO/glass substrates at (**b**) 25 °C, (**c**) 200 °C, (**d**) 250 °C, and (**e**) 300 °C.

The size distribution of the nanoparticles was determined from the SEM micrographs by using particle size histograms, as shown in Figure 4. The average size of the nanoparticles in the FTO/glass substrate was determined to be 237 nm. After the deposition of the nanocomposite thin films, the as-determined nanoparticle sizes were found to be 258, 234, 242, and 247 nm for the thin films deposited at 25 °C, 200 °C, 250 °C, and 300 °C, respectively. We should note that the measured nanoparticle diameters in Figure 4b–e correspond to the FTO grains covered by a thin layer of TiO_2_–SiO_2_, and the increase in diameter after deposition indicates the overlaying of FTO grains.

#### 3.2.2. Energy Dispersive X-ray Analysis

**Figure 5:**
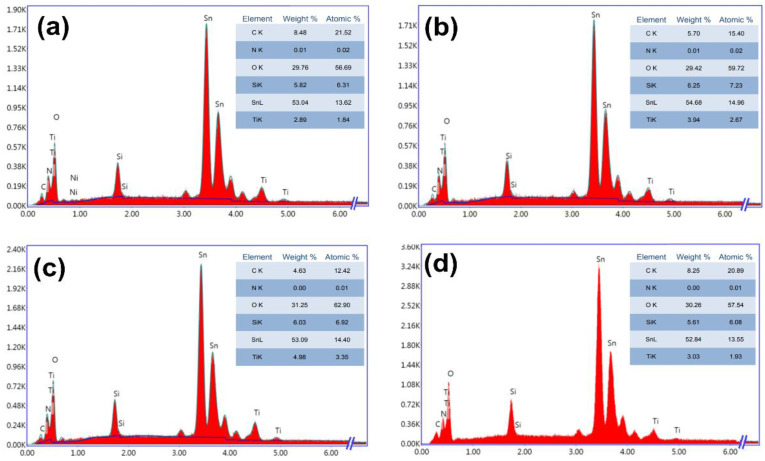
EDX spectra of TiO_2_–SiO_2_ nanocomposite thin films deposited at (**a**) 25 °C, (**b**) 200 °C, (**c**) 250 °C, and (**d**) 300 °C.

Figure 5 shows the EDX spectra of the nanocomposite thin films. As can be seen from the figure, oxygen, silicon, titanium, and tin (originating from the TiO_2_–SiO_2_ thin film/FTO/glass substrate) are the main elements. In addition, carbon was detected in all samples as well as in the sintered target (Figure 1b), originating from the oil contamination of the PLD vacuum system [32], while nitrogen was found as trace. We should note that we could not deduce the structural composition of the thin films from the EDX results and by using Equation (1) because the silicon atomic percentage measured was present in the thin films as well as in the glass substrates. The atomic percentage of tin was approximately the same in all samples, however, the titanium atomic percentage was different, and it depended on the deposition temperature.

**Figure 6:**
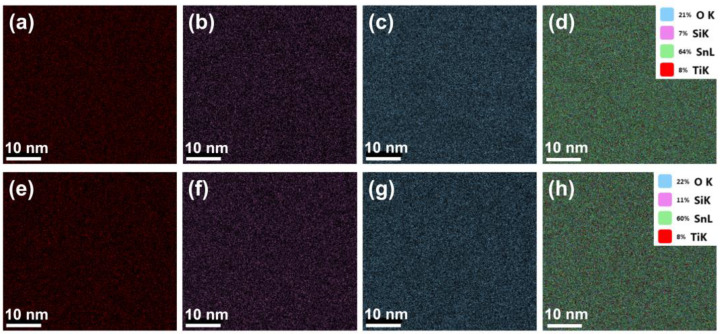
EDX mapping images of (**a**–**d**) TiO_2_–SiO_2_ nanocomposite thin films deposited at 25 °C and (**e**–**h**) TiO_2_–SiO_2_ nanocomposite thin films deposited at 300 °C, showing the distribution of the constituent elements Ti, Si, O, and Sn within the films.

We conducted EDX mapping in order to determine the elements’ distribution within the surfaces of the nanocomposite thin films deposited at 25 °C (Figure 6a–d) and at 300 °C (Figure 6e–h). As illustrated in the same figure, titanium (Figure 6a,e), silicon (Figure 6b,f), and oxygen (Figure 6c,g) were distributed homogeneously along the surface of the films. The tin element observed in Figure 6d,h originated from the FTO/glass substrate. From the same figures, we can deduce that the Ti contents in the TiO_2_–SiO_2_ nanocomposite thin films were the same for thin films deposited at 25 °C and 300 °C. 

#### 3.2.3. Raman Spectroscopy

**Figure 7:**
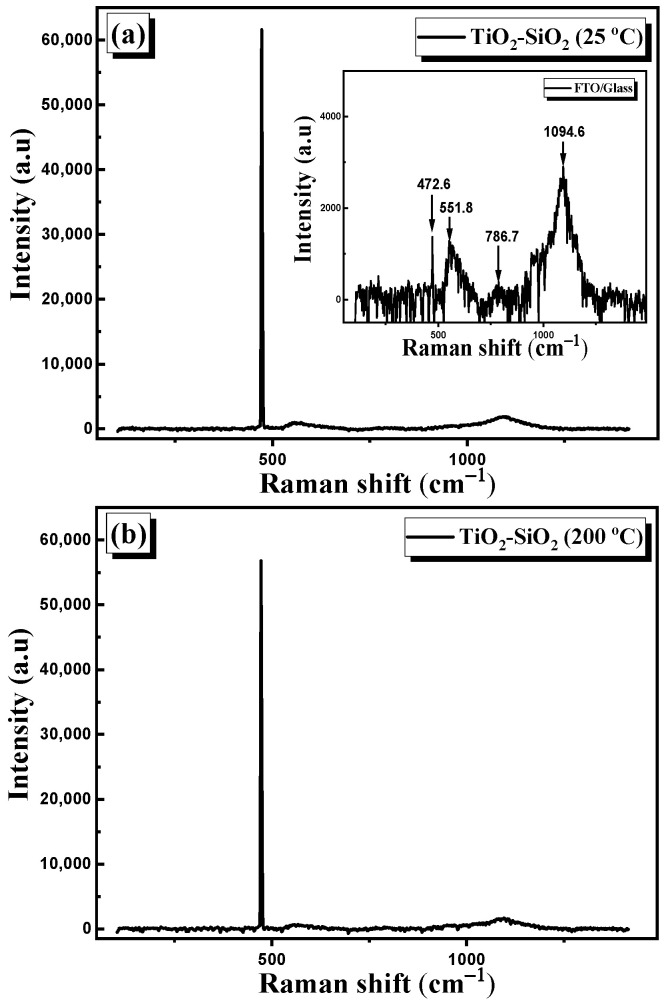

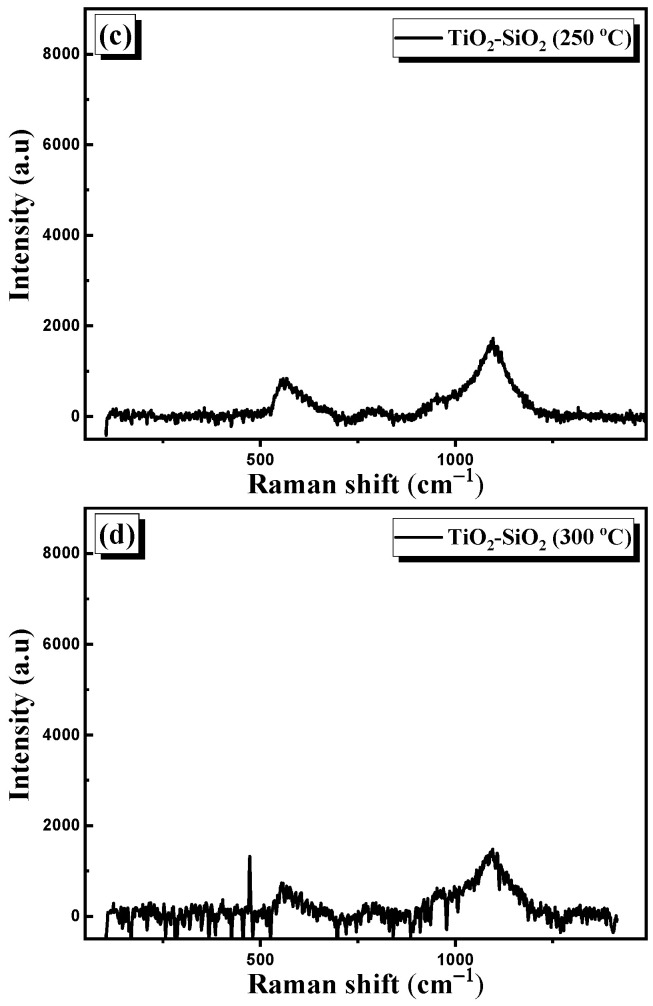
Raman spectra of TiO_2_–SiO_2_ nanocomposite thin films deposited at (**a**) 25 °C, (**b**) 200 °C, (**c**) 250 °C, and (**d**) 300 °C.

Raman spectra of the PLD-deposited nanocomposite thin films are shown in Figure 7. The first observation is that all the peaks detected in the Raman spectra in the sintered TiO_2_–SiO_2_ nanocomposite target (Figure 2a) were absent in the TiO_2_–SiO_2_ nanocomposite films. Raman spectra corresponding to the FTO/glass substrate are shown in the inset of Figure 7a. The substrate exhibits peaks at 472.6, 551.8, 786.7, and 1094.6 cm^−1^. The peak at 472.6 cm^−1^ can be attributed to the E_g_ mode in the FTO, which is related to the oxygen vibration in the oxygen plane [33,34]. The peak around 551 cm^−1^ may be related to the small size effect of the nanoparticles in FTO thin films [34]. Moreover, the peak located at 787 cm^−1^ can be attributed to *B_2g_*s, which is related to the expansion and contraction of Sn–O bonds. Finally, the peak at 1094.6 cm^−1^ can be attributed to the glass substrate [35]. As can be seen in Figure 7a–d, all samples display the same peaks as the substrate, thus indicating that the deposited thin films of TiO_2_–SiO_2_ possess an amorphous structure. 

#### 3.2.4. XRD Characterization

**Figure 8:**
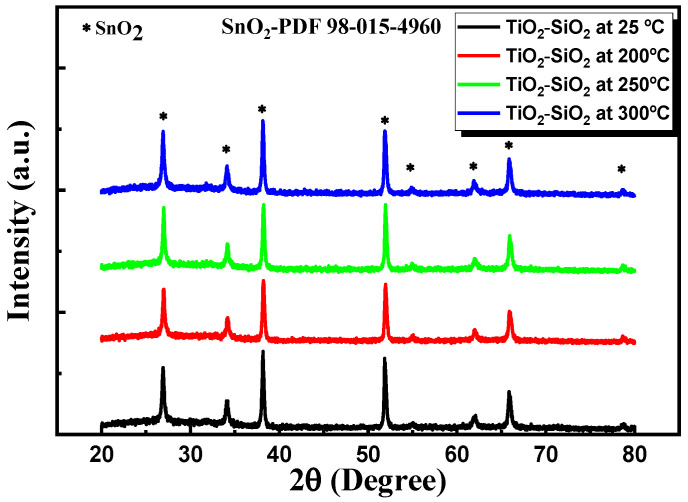
X-ray diffraction patterns of TiO_2_–SiO_2_ nanocomposite thin films.

We carried out an XRD analysis to determine whether any TiO_2_ and SiO_2_ phases had formed in the nanocomposite thin films deposited by PLD. The corresponding recorded XRD patterns are presented in Figure 8. It can be easily observed that all diffractograms reveal a tetragonal SnO_2_ structure related to the FTO/glass substrate, which is in good agreement with JCPDS card SnO_2_—PDF 98-015-4960 [36]. The crystalline structures of TiO_2_ and SiO_2_ could not be assessed from the XRD patterns, likely due to the very thin layer of the nanocomposite and/or due to its amorphous structure. The assumption that the TiO_2_–SiO_2_ nanocomposite nanofilms exhibit an amorphous structure further supports the results revealed by the Raman analysis [Figure 7]. 

#### 3.2.5. Optical Characterization

**Figure 9:**
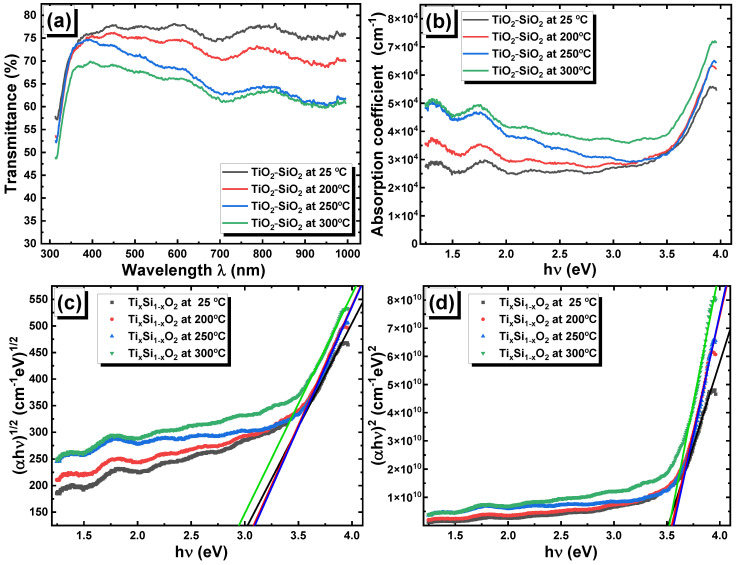
(**a**) Transmittance spectra, (**b**) absorption spectra, (**c**) Tauc plot for indirect optical band gaps, and (**d**) Tauc plot for direct optical band gaps in the TiO_2_–SiO_2_ nanocomposite thin films.

Figure 9a shows the spectral dependence of transmittance (T) in the four samples. As can be seen from the figure, the transmittance decreases in the ultraviolet and the visible ranges as the PLD deposition temperature increases from 25 to 300 °C. The optical absorption coefficient (α) was determined using Equation (2), with the assumption that the reflection in all samples was negligeable:

(2) $$\alpha =-ln\left(T\right)/d$$

In the above equation, *d* represents the thin film thickness. The light absorption was significantly enhanced in the visible region as the PLD deposition temperature increased, as can be seen in Figure 9b, which could be due to the formation of band tail states in the forbidden band gap as the deposition temperature increased.

The absorption coefficient is related to the photon energy (hν) and the optical band gap energy ($E_{g}$) of the nanocomposite thin films, according to the following equation:

(3) $$\alpha =\frac{A(h\nu -E_{g}{)}^{m}}{h\nu }$$

where *h* is the Planck constant, ν is the light frequency, and *A* represents a constant that depends on the transition nature indicated by the value of m. Equation (3) can be simplified as follows:

(4) $$\frac{d\left[\ln(\alpha h\nu )\right]}{d(h\nu )}=\frac{m}{h\nu -E_{g}}$$

The Tauc exponent *m* can have a value of 0.5 for the direct optical bandgap or a value of 2 for an indirect optical band gap [37]. Thus, we drew the graphs ${\left(\alpha h\nu \right)}^{1/2}$ and ${\left(\alpha h\nu \right)}^{2}$ as functions of the photon energy, as shown in Figure 9c,d, respectively. Both the $f\left(h\nu \right)={\left(\alpha h\nu \right)}^{1/2}$ and $f\left(h\nu \right)={\left(\alpha h\nu \right)}^{2}$ plots showed linear dependence as a function of the incidence photon energy in the UV region. The energies of the direct and indirect possible transitions were determined by extrapolating the linear parts of the previously mentioned curves. The indirect optical band gap energies were calculated and found to be *Eg* = 3.00, 3.06, 3.07, and 2.92 eV for thin films deposited at 25 °C, 200 °C, 250 °C, and 300 °C, respectively. The obtained band gap energies are close to those reported in crystalline TiO_2_ [38]. Additionally, the direct optical band gap energies were estimated to be *Eg* = 3.50, 3.53, 3.55, and 3.51 eV for thin films deposited at 25 °C, 200 °C, 250 °C, and 300 °C, respectively. These match with the values of the direct energy band gaps in TiO_2_–SiO_2_ nanocomposite thin films reported by Huong et al. [39].

## 4. Conclusions

A highly nanostructured TiO_2_–SiO_2_ nanocomposite disk-shaped target was fabricated which was comprised of nanoparticles with an average size of 132 nm. TiO_2_–SiO_2_ nanocomposite thin films were deposited successfully on FTO/glass substrates using the PLD technique at different deposition temperatures. The microstructural characterization of the thin films revealed the amorphous structure and highly homogeneous distribution of the nanocomposites within the surfaces. The thin films under study exhibited indirect optical band gap energies ranging from 2.92 to 3.07 eV, while their direct optical band gap energies lay between 3.50 and 3.55 eV. The nanocomposite thin film deposited at 25 °C displayed the best optical performance in terms of transmittance in the visible range, which is very suitable for application as an electron transport layer for low-temperature and flexible perovskite solar cells. This research aims to uncover new insights into the deposition of amorphous TiO_2_–SiO_2_ nanocomposite thin films using the PLD technique for perovskite solar cell technology applications. As a future research objective, we intend to investigate the electrical and transport properties of the TiO_2_–SiO_2_ nanocomposite thin films.

## Data Availability

Data are available from the corresponding author upon the request.

## References

[1] Dette C., Pérez-Osorio M.A., Kley C.S., Punke P., Patrick C.E., Jacobson P., Giustino F., Jung S.J., Kern K. (2014). TiO_2_ anatase with a bandgap in the visible region. Nano Lett..

[2] Sun S., Song P., Cui J., Liang S. (2019). Amorphous TiO_2_ nanostructures: Synthesis, fundamental properties and photocata-lytic applications. Catal. Sci. Technol..

[3] Möls K., Aarik L., Mändar H., Kasikov A., Niilisk A., Rammula R., Aarik J. (2019). Influence of phase composition on optical properties of TiO_2_: Dependence of refractive index and band gap on formation of TiO_2_-II phase in thin films. Opt. Mater..

[4] O’regan B., Grätzel M. (1991). A low-cost, high-efficiency solar cell based on dye-sensitized colloidal TiO_2_ films. Nature.

[5] Zhu W., Zheng G., Cao S., He H. (2018). Thermal conductivity of amorphous SiO_2_ thin film: A molecular dynamics study. Sci. Rep..

[6] Zhang Q., Liu H., Zhao S., Dong W. (2021). Hydrophobic and optical properties of silica antireflective coating prepared via sol-gel method. Mater. Res. Express..

[7] Masuda Y., Jinbo Y., Koumoto K. (2009). Room temperature CVD of TiO_2_ thin films and their electronic properties. Sci. Adv. Mater..

[8] Baek G., Baek J.H., Kim H.M., Lee S., Jin Y., Park H.S., Kil D.S., Kim S., Park Y., Park J.S. (2021). Atomic layer chemical vapor deposition of SiO_2_ thin films using a chlorine-free silicon precursor for 3D NAND applications. Ceram. Int..

[9] Weng X., Fisher P., Skowronski M., Salvador P.A., Maksimov O. (2008). Structural characterization of TiO_2_ films grown on LaAlO_3_ and SrTiO_3_ substrates using reactive molecular beam epitaxy. J. Cryst. Growth.

[10] Shibata H., Kimura S., Takatoh H. (2000). Deposition of SiO_2_ thin films by combined low-energy ion-beam and molecular-beam epitaxial method. Jpn. J. Appl. Phys..

[11] Xu J., Nagasawa H., Kanezashi M., Tsuru T. (2021). TiO_2_ coatings via atmospheric-pressure plasma-enhanced chemical vapor deposition for enhancing the UV-resistant properties of transparent plastics. ACS Omega.

[12] Ryu C.I., Choa S.H. (2020). Evaluation of Argon as a Carrier Gas of Liquid Material Vaporization During the Plasma-Enhanced Chemical Vapor Deposition (PECVD) Silicon Oxide Process. Sci. Adv. Mater..

[13] Devi K.P., Goswami P., Chaturvedi H. (2022). Fabrication of nanocrystalline TiO_2_ thin films using Sol-Gel spin coating technology and investigation of its structural, morphology and optical characteristics. Appl. Surf. Sci..

[14] Wang J., Ran Q., Xu X., Zhu B., Zhang W. (2019). August. Preparation and Optical Properties of TiO_2_–SiO_2_ thin films by Sol-gel Dipping Method. IOP Conf. Ser. Earth Environ. Sci..

[15] Kadri L., Bulai G., Carlescu A., George S., Gurlui S., Leontie L., Doroftei C., Adnane M. (2021). Effect of Target Sintering Temperature on the Morphological and Optical Properties of Pulsed Laser Deposited TiO_2_ Thin Films. Coatings.

[16] Koike R., Suzuki R., Katayama K., Higashihata M., Ikenoue H., Nakamura D. (2022). Formation dynamics of SiO_2_ nanoparticles produced by laser ablation in ambient gases. Appl. Phys. A.

[17] Subedi B., Puli V.S., Boyd I.W., Chrisey D.B., Guo C., Sing S.C. (2021). Pulsed Laser Deposition of Thin Films. Handbook of Laser Technology and Applications.

[18] Haider A.J., Alawsi T., Haider M.J., Taha B.A., Marhoon H.A. (2022). A comprehensive review on pulsed laser deposition technique to effective nanostructure production: Trends and challenges. Opt. Quantum Electron..

[19] Axente E., Socol G. (2022). Pulsed Laser Deposition of Thin Films: Recent Advances and Challenge. Coatings.

[20] Rosales A., Ortiz-Frade L., Medina-Ramirez I.E., Godínez L.A., Esquivel K. (2021). Self-cleaning of SiO_2_–TiO_2_ coating: Effect of sonochemical synthetic parameters on the morphological, mechanical, and photocatalytic properties of the films. Ultrason. Sonochem..

[21] Yang P., Tang Q., He B. (2015). Toward elevated light harvesting: Efficient dye-sensitized solar cells with titanium dioxide/silica photoanodes. RSC Adv..

[22] Zhang L., Hörantner M.T., Zhang W., Yan Q., Snaith H.J. (2017). Near-neutral-colored semitransparent perovskite films using a combination of colloidal self-assembly and plasma etching. Sol. Energy Mater Sol. Cells.

[23] Hodroj A., Chaix-Pluchery O., Audier M., Gottlieb U., Deschanvres J.L. (2008). Thermal annealing of amorphous Ti–Si–O thin films. J. Mater. Res..

[24] Zhao W., Jia H., Qu J., Yang C., Wang Y., Zhu J., Wu H., Liu G. (2022). Sol-gel synthesis of TiO_2_–SiO_2_ hybrid films with tunable refractive index for broadband antireflective coatings covering the visible range. J. Solgel Sci. Technol..

[25] Mitronika M., Villeneuve-Faure C., Massol F., Boudou L., Ravisy W., Besland M.P., Goullet A., Richard-Plouet M. (2021). TiO_2_–SiO_2_ mixed oxide deposited by low pressure PECVD: Insights on optical and nanoscale electrical properties. Appl. Surf. Sci..

[26] Wang J., Bai L., Xiao Y., Luo Y., Zhu B., Zhang W. (2020). Synthesis, wettability and optical properties of Ti_x_O_2_– Si_(1-x)_O_2_ composite thin films. IOP Conf. Ser. Mater. Sci. Eng..

[27] Kunti A.K., Chowdhury M., Sharma S.K., Gupta M., Chaudhary R.J. (2017). Influence of O_2_ pressure on structural, morphological and optical properties of TiO_2_–SiO_2_ composite thin films prepared by pulsed laser deposition. Thin Solid Film..

[28] Hardiansyah A., Budiman W.J., Yudasari N., Isnaeni K.T., Wibowo A. (2021). Facile and green fabrication of microwave-assisted reduced graphene oxide/titanium dioxide nanocomposites as photocatalysts for rhodamine 6G degradation. ACS Omega.

[29] Zhang Q., Ma L., Shao M., Huang J., Ding M., Deng X., Wei X., Xu X. (2014). Anodic oxidation synthesis of one-dimensional TiO_2_ nanostructures for photocatalytic and field emission properties. J. Nanomater..

[30] Jiang Z., Yang D., Wang N., Zhang F., Zhao B., Tan S., Zhang J. (2013). Inverted polymer solar cells with TiO_2_ electron extraction layers prepared by magnetron sputtering. Sci. China Chem..

[31] Su T.S., Hsieh T.Y., Hong C.Y., Wei T.C. (2015). Electrodeposited ultrathin TiO_2_ blocking layers for efficient perovskite so-lar cells. Sci. Rep..

[32] Jackson B.D. (1997). Pulsed-Laser Deposition of Silicon Dioxide Thin-films Using the Molecular Fluorine Laser. Master’s Thesis.

[33] Wang X., Wang X., Di Q., Zhao H., Liang B., Yang J. (2017). Mutual effects of fluorine dopant and oxygen vacancies on structural and luminescence characteristics of F doped SnO_2_ nanoparticles. Materials.

[34] Wan W., Li Y., Ren X., Zhao Y., Gao F., Zhao H. (2018). 2D SnO_2_ nanosheets: Synthesis, characterization, structures, and excellent sensing performance to ethylene glycol. Nanomaterials.

[35] Shcheblanov N.S., Mantisi B., Umari P., Tanguy A. (2015). Detailed analysis of plastic shear in the Raman spectra of SiO_2_ glass. J. Non-Cryst. Solids.

[36] Asl H.Z., Rozati S.M. (2019). High-quality spray-deposited fluorine-doped tin oxide: Effect of film thickness on structural, morphological, electrical, and optical properties. Appl. Phys. A.

[37] Makuła P., Pacia M., Macyk W. (2018). How to correctly determine the band gap energy of modified semiconductor photocatalysts based on UV–Vis spectra. J. Phys. Chem. Lett..

[38] Eddy D.R., Permana M.D., Sakti L.K., Sheha G.A.N., Solihudin H.S., Takei T., Kumada N., Rahayu I. (2023). Heterophase Polymorph of TiO_2_ (Anatase, Rutile, Brookite, TiO_2_ (B)) for Efficient Photocatalyst: Fabrication and Activity. Nanomaterials.

[39] Huong H.T., Nhu T.T.Q., Nang H.X., Tuan P.A., Huy P.T. (2023). Super-hydrophilic Ce^3+^-doped TiO_2_-SiO_2_ nanocomposite thin films with high optical transmittance by a simple spin-coating method. Thin Solid Film..

